# Thermodynamic Properties of Polyethylene Predicted from Paraffin Data

**DOI:** 10.6028/jres.067A.023

**Published:** 1963-06-01

**Authors:** Martin G. Broadhurst

## Abstract

Thermodynamic data on the *n*-paraffins from *n*-C_6_H_14_ through *n*-C_18_H_38_ have been used to obtain values for the specific heat, entropy, enthalpy, and Gibbs free energy of a large, ideal CH_2_-chain crystal from 0 to 420 °K and of the liquid above 200 °K. Analytical expressions are given for the properties of the crystal and liquid above 200 °K. For the crystal, a modified Einstein function was used to adjust the melting temperature to 414.3 °K. Values between 975 and 1025 cal/mole for the heat of fusion were found to be the ones most consistent with the data. Comparison of the results with polyethylene data shows reasonable agreement.

## 1. Introduction

This paper concerns the empirical determination of accurate values for the thermodynamic properties of ideal CH_2_-chain crystal^1^ and liquid in the temperature range from 0 to 420 °K and 200 to 420 °K respectively, using published data on pure *n*-paraffins. This analysis is intended to circumvent the inherent difficulties involved in measuring polyethylene directly since (so far) even the most carefully crystallized polyethylene samples are not large, well defined CH_2_-chain systems, and measurements necessarily reflect the nonnegligible effects of chain folds and “amorphous” inclusions. Wunderlich [1],^2^ realizing this problem, has extrapolated specific heats to 100 percent crystallinity to obtain the crystalline CH_2_ contribution to the specific heat. This method is presently limited in accuracy by a scarcity of data and variations in the chemical structures (e.g., degree of branching and molecular weight) which exist between samples of different crystallinity. Even ideally, if one had sufficient data on several polyethylene samples which differed in percent crystallinity, the extrapolated results might still reflect the presence of chain folded lamellar surfaces, unless the lamellar thicknesses were properly taken into account.

The use of *n*-paraffin data to determine CH_2_ properties offers the advantages that the *n*-paraffins can be made highly pure and crystallized to a high degree of perfection. Very carefully measured specific heat data are available for pure *n*-paraffins from *n*-C_6_H_14_ through *n*-C_18_H_38_ (see following section). It has been reported that the molar specific heat (*C_p_*),^3^ enthalpy (*H*), entropy (*S*), and Gibbs free energy (*F*) of the solid *n*-paraffins appear to be linear with chain length at constant temperature and crystal phase [2–4]. An obvious interpretation of this observation is that each CH_2_ unit in the chain can be thought of as contributing a certain number of vibrational modes to the crystal without changing the frequency spectrum, and that this contribution is independent of the length of the chain in which the unit is located, so long as the local crystal structure remains unchanged. Accordingly, the properties of a CH_2_ unit should be the same in a large ideal —CH_2_— crystal as in the *n*-paraffins. The major limitation to the *n*-paraffin determination of the crystalline CH_2_ properties are the deviations from linearity with chain length which become appreciable at temperatures near the *n*-paraffin melting points.

For the liquid phase, there is a slight but real departure from linearity which will necessitate special treatment in the determination of the liquid phase properties.

The approach used below will be: (a) To establish the best values for *C_p_*, *H*, *S*, and *F* of a —CH_2_— unit in both the liquid and solid phases over as wide a range of temperatures as possible, (b) to establish a consistent analytical representation of the above data from 200 to 420 °K, and (c) to compare the results with actual data on polyethylene and examine the discrepancies between the predicted and observed properties.

## 2. Source of Data

The *n*-paraffin data used in this study were furnished by the United States Bureau of Mines, Bartlesville, Okla., in the form of tables of values for *C_p_*, *H-H*_0_, *S*, and *F-H*_0_ (*H*_0_ is the enthalpy at 0 °K). Their work on *n*-C_6_H_14_, *n*-C_7_H_16_, and *n*-C_8_H_18_ through *n*-C_16_H_34_ is presented, together with details of sample purity, measurement techniques, and thermodynamic calculations, in references [5, 6, and 7] respectively, and their work on *n*-C_17_H_36_ and *n*-C_18_H_38_ is as yet unpublished. The data on *n*-C_7_H_16_, *n*-C_17_H_36_ and *n*-C_18_H_38_ covered the temperature range from 0 to 370 °K, and data for the remaining paraffins extended from 0 to 320 °K.

## 3. CH_2_ Structures

The even *n*-paraffins^4^ from C_6_H_14_ through C_18_H_38_ are triclinic at all temperatures up to the melting point. The odd *n*-paraffins 4 from C_11_H_24_ through C_17_H_36_ are orthorhombic at all temperatures up to within 15 degrees of their melting points but undergo a solid-solid transition prior to melting. The orthorhombic CH_2_ structure of the odd paraffins appears to be identical to the CH_2_ structure of polyethylene, and is assumed to be identical to the structure of the ideal CH_2_-chain crystal whose properties we are predicting. Although one would not expect the data for the triclinic structure of the even *n*-paraffins to be relevant to this work, it was found that the data obtained from the even *n*-paraffins varied only slightly from that of the odd *n*-paraffins.

In the liquid state one expects and finds no even-odd differences among the paraffins. The absolute entropies of all 13 paraffins examined here, when plotted against chain length, lie on the same, nearly linear curve and the enthalpies and free energies show a similar behavior if one takes into account the differences in zero point enthalpies between the different solid structures. For instance, the enthalpy which had to be added to *F* and *H* for the even paraffins in order to have the liquid phase values of *F* and *H* for both even and odd paraffins lie on the same nearly linear curve, when plotted versus *n*, was found to be 370±10 cal/mole of paraffin molecules. This zero point energy difference is independent of chain length since it results entirely from the differences in packing of the chain end groups, with the triclinic phase allowing the lowest energy packing.^5^

Whereas the solid phase of very linear, high molecular weight polyethylene contains a significant volume of chain folds which cause deviations from the ideal crystalline CH_2_ specific heats, the liquid state should present no such difficulties. Specific heat measurements should not be able to detect the small percentage of chain ends and branches present in the liquid. Therefore, the specific heats and absolute entropies calculated here from the liquid *n*-paraffin data should be close to the actual measured values for polyethylene, and a comparison of predicted and experimental properties of the liquid provides an experimental test of this work. The liquid paraffin data should not, however, be expected to reflect the presence of a glassy state even well below the temperature where the ideal CH_2_-chain liquid might become a glass.

## 4. Analysis of the Paraffin Data

Table 1 contains a sample of the thermodynamic data as received for the solid and liquid phases at representative temperatures.

For the solid at a given temperature, the differences between the thermodynamic values of consecutive even and consecutive odd compounds was computed and these differences were averaged over the whole range of compounds for which data were available at that temperature. The averages for the even and odd paraffins were found to be very similar, and the combined average for both even and odd compounds was divided by two to obtain the desired thermodynamic value per mole of CH_2_ units.

At the higher temperatures, where the shorter paraffins are liquid, the accuracy of the computations decreases due to the decreased range of data and the premelting^6^ increase in the specific heat that begins about 50 degrees below the melting point and which can be seen in the data at 200 °K in table 1. It is probable that above 200 °K, the values calculated by the above procedure will be somewhat smaller than the correct values.

For the liquid phase data, the range of temperatures amenable to computation was limited at low temperatures by the decrease in the number of liquid samples and at high temperatures (370 °K) by the termination of experimental measurements. The most reliable values are those around 300 °K where most data exist.

The differences between the thermodynamic values for consecutive even and odd paraffins are not quite as constant for the liquid as for the solid as can be seen in table 1, and an appropriate extrapolation of these differences to *n*= ∞ was considered to be preferable to using a simple average. When plotted versus 1/*n*, the differences for the liquid were found to be linear within experimental error. For *F*, *H*, and *S*, the best linear extrapolation to 1*/n *= 0 was graphically determined, and the resulting intercept was chosen as the desired thermodynamic value. For *C_p_*, this 1*\n* extrapolation gave values which exhibited excessive scatter. It was found that this scatter could be reduced by integrating the *C_p_* data, extrapolating the integrated values to 1/*n*=0 and then differentiating the extrapolated integrated values to obtain the desired extrapolated *C_p_* values. In practice, this process merely involved taking differences between the consecutive experimental *H* values in table 2 and dividing by 10°. For example, *C_p_* (per mole of CH_2_ units) at 305 °K was taken to equal *H* (per mole of CH_2_ units) at 310 °K minus *H* (per mole of CH_2_ units) at 300 °K divided by 10°. The same procedure was followed (though mathematically, less precisely) using the TS data, in order to increase the number of available *C_p_* values.

**Figure 1:**
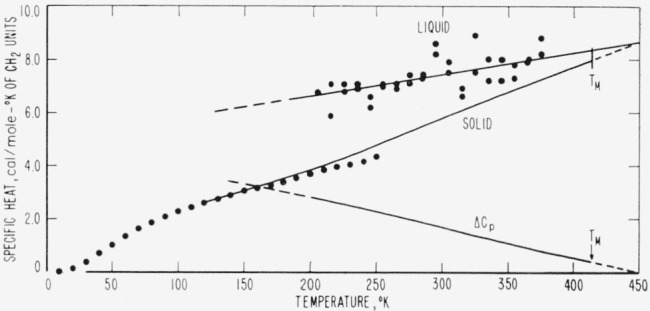
Predicted specific heats, *C_p_*, of an ideal *CH*_2_–chain crystal and liquid as a function of temperature. The closed circles represent values calculated from n-paraffin data, samples of which are listed in table 1, and the solid curves were calculated from the equations in table 3.

The results of the above determinations are listed in table 2 and are represented graphically as closed circles in figures 1 through 4. The large number of digits given in the calculated value columns in table 2 is not intended to indicate significant figures but is included only for internal consistency.

## 5. Determination of Analytical Expressions

Linear least squares fits of the specific heats for the solid above 150 °K and specific heats for the liquid above 200 °K as a function of temperature were carried out. The calculated standard deviations were 0.15 c l/mole-°K for the solid (39 data points) and 0.84 cal mole-°K for the liquid (36 data points). Integrations of *C_p_* and *C_p_/T* were then carried out and the average values of the constants of integration were determined from the entropy and enthalpy data. The resulting functions fit the *H* and *S* data for the solid above 200 °K with a standard deviation of 2 cal/mole and 0.01 cal/mole-°K, respectively, and for the liquid above 200 °K with a standard deviation of 6 cal/mole and 0.02 cal/mole-°K, respectively. The Gibbs free energy was computed directly from *H* and *S* and was found to fit the free energy data above 200 °K with a standard deviation of 4 cal/mole for the solid and 9 cal/mole for the liquid.

By using the calculated analytical expressions to extrapolate up to the temperature where the free energies for the solid and liquid become equal, it was found that the predicted melting point was 397.4 °K and the predicted heat of fusion was 1132 cal/mole. Since the predicted melting point is 4 percent below its most probable value of 414.3 °K [4] and since the predicted heat of fusion seems unreasonably high, the assumption of a linear specific heat for the solid above 200 °K was concluded to be inadequate for the representation of the known data. Hence, a correction to the specific heat for the solid was sought that would decrease the free energy of the solid by a sufficient amount to increase the melting point to its previously determined most probable value, and at the same time the correction was desired to have as little effect as possible on the linear thermodynamic functions for the solid below 300 °K where a good fit of the data was already obtained.

Fortunately, a correction of the type implied here by the *n*-paraffin data has the same form as the specific heat contributions due to several independent CH_2_ vibrations which become important above 250 °K [8, 9]. These vibrations are well represented by a summation of Einstein functions of the form $C_{v}=\sum_{i}\left(R\theta_{i}^{2}/T^{2}\right)\left[e^{\theta_{i}/T}/{\left(e^{\theta_{i}/T}-1\right)}^{2}\right]$. For our use, we have preserved the form of this function but have applied it to *C_p_* rather than *C_v_* and have made the further approximations that we can use a single term to replace the summation of several terms, and that *θ*>>*T* in the range of interest. That is, we shall assume a correction to *C_p_* of the form,

$${\bar{C}}_{p}=\left(\bar{n}R{\bar{\theta }}^{2}/T^{2}\right)\;\exp\;\left(-\bar{\theta }/T\right),$$ (1)

where the bar indicates an “effective’’ quantity which is expected to lie somewhere between the maximum and minimum quantities in the above expression for *C_v_.* The effective number of independent CH_2_ modes is represented by $\bar{n}$, *R* is the molar gas constant, $\bar{\theta }=h\bar{v}/k$ is the characteristic temperature, *h* is Planck’s constant, $\bar{v}$ is the effective frequency of vibration, *k* is Boltzmann’s constant, and *T* is the absolute temperature.

It is convenient to replace the two parameters $\bar{n}$ and $\bar{\theta }$ in eq (1) by two different parameters, *F_s_* and *H_s_*, the corrections to the free energy and enthalpy at *T_M_* needed to give the proper melting- point and heat of fusion. This replacement is accomplished by integrating eq (1) to find the corresponding $\bar{H}$ and $\bar{F}$ and then solving the equations $H_{s}=\bar{H}$ at *T = T_M_* and $F_{s}=\bar{F}$ at *T= T_M_* for $\bar{\theta }$ and $\bar{n}$. The result is $\bar{n}=\left(F_{s}/RT_{M}\right)\;\exp\;\left(H_{s}/F_{s}\right)$ and $\bar{\theta }=T_{M}H_{s}/F_{s}$ If we assume *T_M_*=414.3 °K[4] then one finds that *F_s_* must equal −48.80 cal/mole (calculated from the uncorrected Δ*F* at 414.3 °K) and *H_s_* must equal (1162.10 − Δ*h_f_*) cal/mole where Δ*h_f_* is the heat of fusion at *T_M_*=414.3 °K. We have here chosen a value of Δ*h_f_* = 1000 cal/mole as reasonable, which choice gives $\bar{\theta }=1376$ and $\bar{n}=1.64$.

The resulting analytical expressions for the thermodynamic funtions of the CH_2_-chain crystal and liquid above 200 °K are shown in table 3, and the calculated values are compared to the experimental values in table 2.

## 6. Discussion

### 6.1. Reliability of the Results

It is important to have some idea of how accurately the results listed in table 2 represent the properties of an ideal CH_2_-chain compound. The specific heats below 150 °K and *H*, *S*, and *F* below 200 °K are probably accurate to ±1 percent judging from the consistency of the averaged values (examples in table 1) and the estimate of better than 1 percent accuracy for the original data. At 200 °K the effects of premelting can be seen in table 1 as a reduction in the first two specific heat differences and the first *H, S*, and *F* differences. One is increasingly uncertain above 200 °K about the correctness of the averaged data values, and thus the calculated values are to be preferred at the higher temperatures. All reasonable attempts to alter the results by starting with different initial linear and quadratic specific heat functions did not change the predicted melting point by more than 2 degrees or the predicted heat of fusion by more than 5 cal/mole. Nor did different assumed forms of the correction term make a significant difference in the predicted thermodynamic functions. The results were found to be quite insensitive to artifacts in the data or initial assumptions. This insensitivity, while giving one confidence in the preducted values, greatly reduces the importance which one can attribute to the values of $\bar{\theta }$ and $\bar{n}$ in eq (1).

The most uncertain and sensitive parameter in the calculations is the heat of fusion, Δ*h_f_.* The best current experimental estimate of Δ*h_f_* for polyethylene by the diluent method is 970 cal/mole [10]. A reasonable calculation shows that for the ideal CH_2_-chain crystal, Δ*h_f_* can be expected to be higher than for 100 A thick lamellar polyethylene by 2 to 3 percent. From our work, Δ*h_f_* = 975.9 appears to be a minimum value since any lower value results in the solid specific heat exceeding the liquid specific heat below the melting point. As Δ*h_f_* is increased above its minimum value, the deviation between the calculated and experimental values in the 200 to 290 °K range increases, and the values of $\bar{\theta }$ and $\bar{n}$ in eq (1) decrease from 1580 °K and 2.70 for Δ*h_f_* =976 to 1376 °K and 164 for Δ*h_f_* = 1000 cal/mole and 1164 °K and 0.98 for Δ*h_f_* =1025. If Δ*h_f_* is as large as 1025 cal/mole, both the deviation from the experimental data and the values of $\bar{\theta }$ and $\bar{n}$ seem unreasonable. Thus it appears likely that the correct value of Δ*h_f_* lies within ±2.5 percent of the 1000 cal/mole assumed in this analysis.

The ±2.5 percent error in Δ*h_f_* causes an uncertainty in *H* and *S* for the solid which decreases rapidly with temperature from about 1 percent at 400 °K to a negligible error at 300 °K. The effect of the uncertainty in Δ*h_f_* on the specific heat is illustrated in figure 5.

For the liquid phase, the standard deviations of the data from the calculated curve have been given in the previous section and amount to roughly 10 percent for *C_p_*, 2 percent for *F*, and 0.3 percent for *II* and *S.* Hence, it seems reasonable to expect the calculated *H* and *S* values to be accurate to within ±2 percent. A comparison with polyethylene liquid data in the next section supports an estimate of ±2 percent reliability for the liquid *C_p_* values as well as the *H* and *S* values.

### 6.2. Comparison With Experiment

Figure 5 shows a comparison of the ideal specific heat predicted here from *n*-paraffin data with that predicted by Wunderlich [1] from polyethylene data and with actual experimental values for crystalline Marlex 50 as reported recently by Passaglia and Kevorkian [9] and Dainton, Evans, Hoare, and Melia [11]. The specific heat values predicted here are appreciably lower than the polyethylene values up to 80 °K, slightly larger from 80 to 140 °K (this range varies somewhat with different data) and again smaller at higher temperatures. The smoothness (smaller variations in the slope) of the experimental *C_p_* curve relative to the predicted curve below room temperature indicates that the ideal vibrational frequency distribution is broadened and the characteristic temperature is lowered in actual specimens of polyethylene—a result attributable to imperfections. Similar behavior was noted during this study in *C_p_* data for a sample of *n*-C_32_H_66_ and a less pure sample of *n*-C_33_H_68_. Whereas the C_33_ specific heats were nearly linear with temperature from 90 to 180 °K, the C_32_ specific heats were markedly sigmoidal in the same temperature range with values below those of C_33_ at high and low temperatures and above those of C_33_ at temperatures between 100 and 150 °K. Liquid polyethylene specific heat data [9], shown as squares in figure 5, agree to within 1 percent with the predicted liquid *C_p_* curve. The enthalpies of the liquid reported by Passaglia and Kevorkian are smaller than predicted by less than 2 percent and the entropies of the liquid are larger than predicted by less than 2 percent. These agreements are considered satisfactory.

Wunderlich’s predicted *H* and *S* values for the solid at 300 °K agree to within 2 percent with those predicted from the *n*-paraffins. The predicted specific heats differ somewhat as shown by the dashed curve in figure 5. Dainton, Evans, Hoare, and Melia [11] used an extrapolation technique to obtain a value for the ideal entropy of polyethylene at 25 °C of 5.5 cal/mole °K in favorable agreement with 5.6 cal/mole °K predicted here.

One important result of this work concerns the thermodynamic crystallinity scale [i.e., the calculation of the crystalline fraction of a sample from the equation χ=[H (liquid) -H(sample)]/[H liquid)-H (crystal)]. The values of H (liquid) -H (crystal) calculated in this study are significantly larger than those given by Wunderlich [1], but both the crystalline and liquid enthalpies are smaller, probably because of a zero point enthalpy difference. Such a zero point enthalpy difference is expected if one assumes that the polyethylene extrapolation pertains to an ideally perfect chain folded lamellar crystal rather than the ideally perfect, large nonfolded crystal associated with the paraffin extrapolation. Whereas both types of perfect crystals would have the same zero point entropy (equal to zero by the third law), the zero point enthalpies would be different. It is interesting to note that the Marlex 50 sample measured by Passaglia and Kevorkian [9] has a volume crystallinity of 80.0 percent and an enthalpy crystallinity of 93.8 percent as determined from the values of Wunderlich and Dole [12], and using enthalpy values from this work, 97 percent or 93.5 percent, depending upon whether one uses the solid at 0 °K or the liquid above *T_M_* as the reference state. The zero point enthalpy correction which had to be added for the latter calculation was 40 cal/mole of folds. The fact that thermodynamic crystallinities are generally higher than volume crystallinities is consistent with the assumption that much of the imperfection in highly crystalline polyethylene samples is unoccupied volume (such as might be expected to exist between lamellar fold surfaces.) Volume measurements would be sensitive to these defects whereas enthalpy measurements would not.

### 6.3. Observations and Suggestions

**Figure 2:**
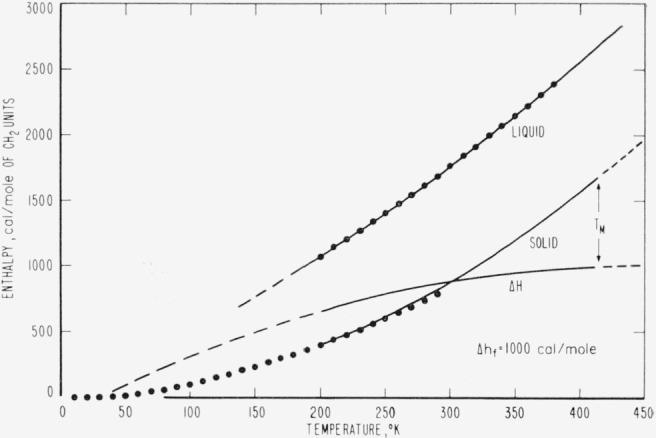
Predicted enthalpies (*H*-*H*_0_), of an ideal *CH*_2_-chain crystal and liquid as a function of temperature. The closed circles represent values calculated from n-paraffin data, samples of which are listed in table 1, and the solid curves were calculated from the equations in table 3.

**Figure 3:**
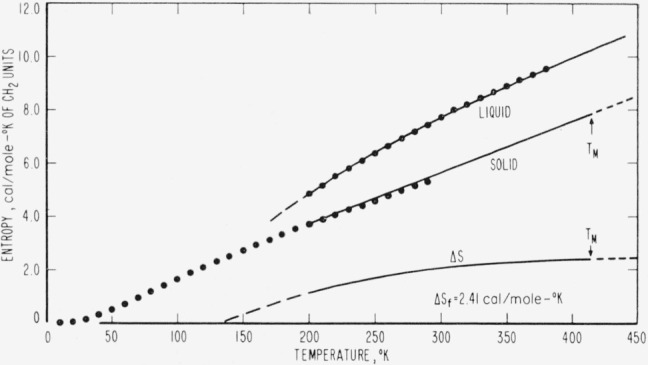
Predicted entropies, *S*, of an ideal *CH*_2_-chain crystal and liquid as a function of temperature The closed circles represent values calculated from n-paraffin data, samples of which are listed in table 1, and the solid curves were calculated from the equations in table 3.

**Figure 4:**
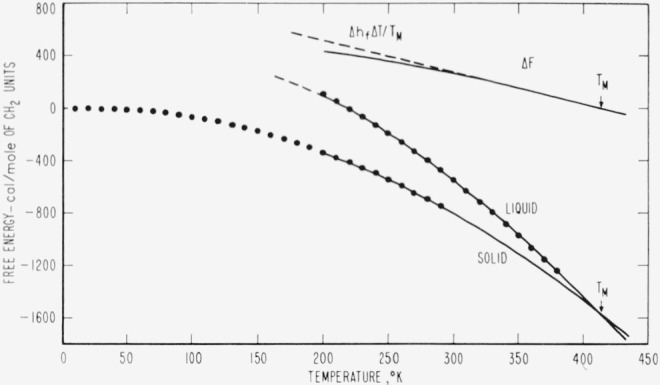
Predicted Gibbs free energies (*F*-*H*_0_) of an ideal *CH*_2_-chain crystal and liquid as a function of temperature. The closed circles represent values calculated from *n*-paraffin data, samples of which are listed in table 1, and the solid curves were calculated from the equations in table 3.

**Figure 5:**
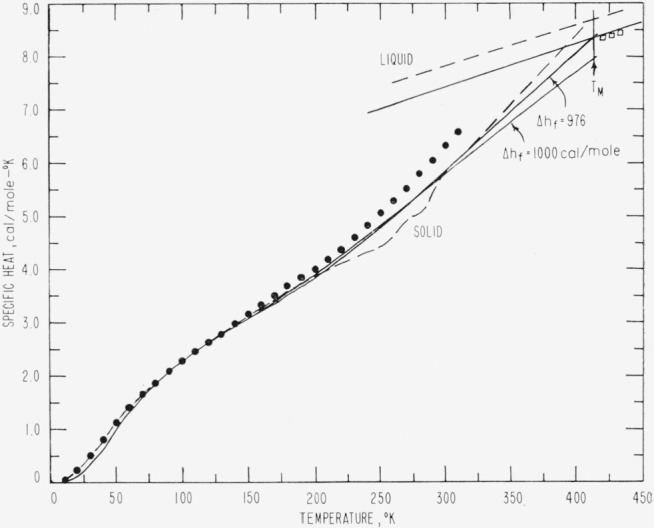
The predicted specific heats, *C_p_*, of an ideal *CH*_2_-chain crystal and liquid (solid curves) for two assumed heats of fusion, compared to the extrapolated values given by Wunderlich for completely crystalline polyethylene (dashed curve) and the polyethylene data of Dainton, Evans, Hoare, and Melia (closed circles) and Passaglia and Kevorkian (open squares).

The thermodynamic properties represented in figures 1–4 exhibit several unusual features that result from the high specific heat of the solid at high temperatures (nearly equal to that of the liquid at the melting temperature). The difference (Δ*H*) between the enthalpies of the solid and liquid is quite constant in the melting region and the tendency of Δ*H* to become negative at low temperatures is not pronounced. Even though Δ*S* appears to tend toward zero between 100 and 150 °K, the necessity for polyethylene to form a glass to maintain a positive Δ*H* and Δ*S* is not as obvious as in most substances. It seems likely that the discontinuity in the Δ*H* versus temperature curve at the glass transition would be so small that it would be difficult to detect. In accord with the above, Δ*F* is well approximated by Δ*h_f_*Δ*T/T_M_* over an unusually large range of temperatures. This simple linear approximation to Δ*f* is in error by only 1.5 percent for Δ*T*=50 degrees and by less than 5 percent for Δ*T*= 100 degrees. One would expect Δ*f* to be much less linear than this for most substances [12]. From the above observations, it is not surprising that there is very little indication of a glass transition in the thermodynamic data for linear polyethylene.

There is no indication in the data derived from the liquid paraffins above 200 °K of the existence of the glassy state in polyethylene, and yet the glass is generally considered to be the stable amorphous state in polyethylene below about 237 °K [1]. One may conclude that the glass transition temperature is chain length dependent and that the glass becomes stable with respect to the supercooled liquid above 200° only for chain lengths greater than some minimum value. The only way one could use the *n*-paraffins for predicting the properties of the polyethylene glassy state would be to have data on the glassy state of the *n*-paraffins.

As shown in table 1, the incremental increase per CH_2_ in the *n*-paraffin thermodynamic properties is not as constant in the liquid state as in the solid. This result seems consistent with the following argument. In the solid, even for chains with less than 10 CH_2_ units, the intermolecular forces are strong enough compared to the intramolecular forces to isolate the motions of a central CH_2_ unit from those of the chain ends. In the liquid the intermolecular forces are weaker and less regular so that the CH_2_ behavior is sensitive to chain end effects. The nonlinearity of the incremental increase per CH_2_ in the liquid thermodynamic properties with reciprocal chain length possibly indicates the presence of “entire chain” vibrational modes in the liquid even for very long chain lengths.^7^

The departure of the predicted specific heat from that measured for polyethylene in the 10 to 80 °K temperature range should be investigated further. Since all polyethylenes so far measured seem to show the same higher than predicted specific heats in this range, the anomaly would seem to be associated with some nonideal feature which all samples have in common—e.g., chain folded surfaces. It would be interesting to examine the changes in specific heat of polyethylene with lamellar thickness to see if the discrepancy could be resolved. It might also be useful to attempt to analyze the data in this paper in the manner reported recently by Wunderlich [8]. It is desirable to have more *n*-paraffin specific heat data for longer chain lengths and higher temperatures in order to increase the accuracy and temperature range of the predicted higher temperature solid and liquid thermodynamic values.

## Figures and Tables

**Table 1:** Sample data and calculations

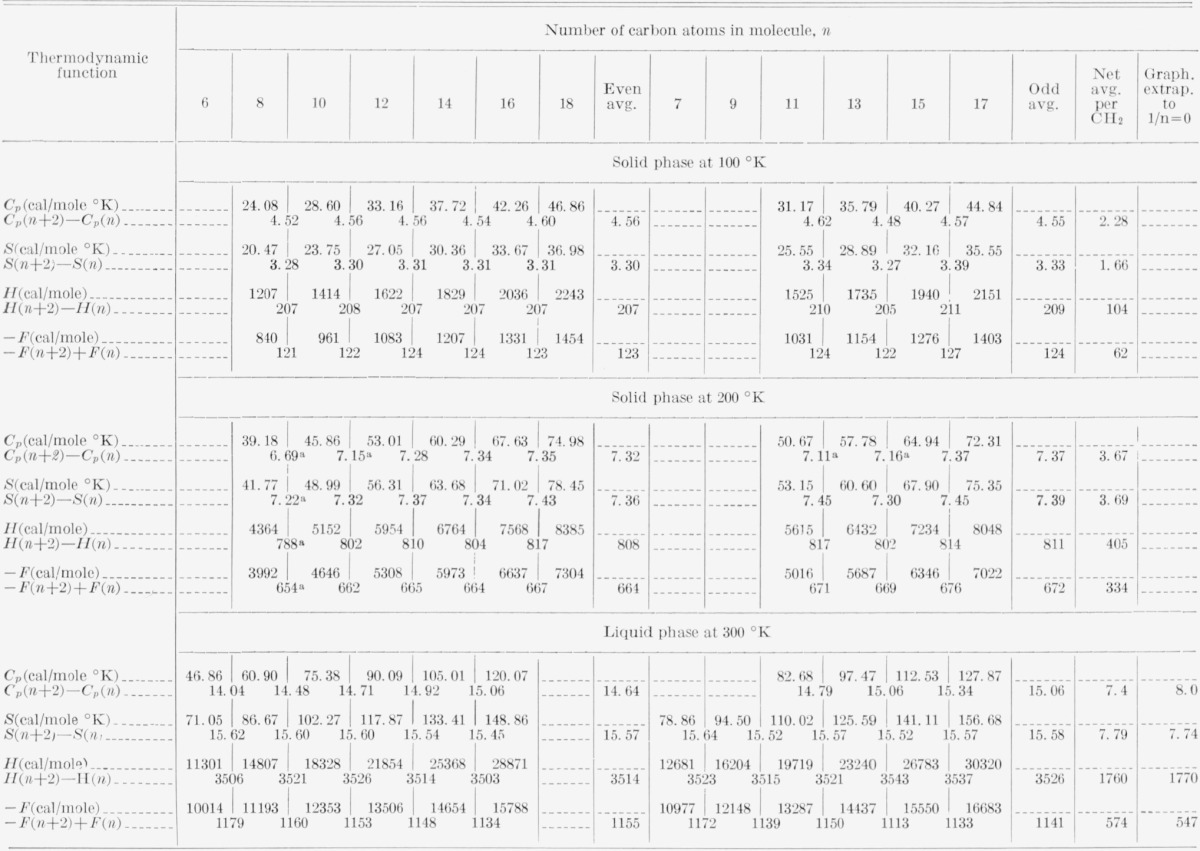

a These values are low because of premelting and were omitted from the calculations.

**Table 2:** Values of the molar specific heats (*C_p_*) enthalpies (*H*–*H*_0_), entropies (*S*) and Gibbs free energies (*F*–*H*_0_) of ideal *CH*_2_ chain crystal and liquid as derived from n-paraffin data (experimental values) and as calculated using the equations in table 3

Temperature	*C_p_* (cal/mole °K)		*H–H*_0_(cal/mole)		*S*(cal/mole °K)		–(*F–H*_0_) (cal/mole)	
	exp.	calc.	exp.	calc.	exp.	calc.	exp.	calc.
	
	Solid phase							
*°K*								
10	0.025	…….	0.06	…….	0.01	…….	0.02	…….
20	.15	…….	.85	…….	.05	…….	.30	…….
30	.39	…….	3.50	…….	.16	…….	1.34	…….
40	.70	…….	9.00	…….	.32	…….	3.7	…….
50	1.03	…….	17.8	…….	.51	…….	7.8	…….
60	1.34	…….	29.5	…….	.73	…….	14.0	…….
70	1.63	…….	44.6	…….	.95	…….	22.4	…….
80	1.88	…….	62.0	…….	1.19	…….	33.2	…….
90	2.10	…….	82.0	…….	1.43	…….	46.2	…….
100	2.28	…….	104	…….	1.66	…….	61.7	…….
110	2.45	…….	128	…….	1.88	…….	79	…….
120	2.60	…….	153	…….	2.10	…….	99	…….
130	2.75	…….	180	…….	2.32	…….	121	…….
140	2.89	…….	208	…….	2.52	…….	146	…….
150	3.05	3.02	237	…….	2.73	…….	171	…….
160	3.16	3.17	268	…….	2.92	…….	200	…….
170	3.28	3.32	300	…….	3.12	…….	230	…….
180	3.40	3.48	334	…….	3.31	…….	263	…….
190	3.52	3.65	369	…….	3.51	…….	297	…….
200	3.67	3.8572	405	405.88	3.69	3.7201	334	338.14
210	3.81	4.0378	441	445.35	3.87	3.9127	371	376.30
220	3.95	4.2224	479	486.64	4.05	4.1046	410	416.39
230	4.05	4.4107	519	529.78	4.23	4.2963	453	458.39
240	4.16	4.6028	562	574.85	4.41	4.4881	496	502.31
250	4.32	4.7975	603	621.84	4.58	4.6799	541	548.15
260		4.9936	647	670.76	4.77	4.8717	590	595.90
270		5.1924	691	721.70	4.94	5.0640	643	645.59
280	…….	5.3909	742	774.57	5.13	5.2562	695	697.18
290	…….	5.5838	790	829.37	5.30	5.4484	748	750.67
300	…….	5.7898	…….	886.34	…….	5.6416	…….	806.15
310	…….	5.9909	…….	945.19	…….	5.8345	…….	863.15
320	…….	6.1851	…….	1005.91	…….	6.0272	…….	922.79
330	…….	6.3836	…….	1068.84	…….	6.2208	…….	983.51
340	…….	6.5795	…….	1133.64	…….	6.4142	…….	1047.20
350	…….	6.7734	…….	1200.32	…….	6.6075	…….	1112.31
360	…….	6.9629	…….	1268.71	…….	6.7999	…….	1179.27
370	…….	7.1553	…….	1339.47	…….	6.9939	…….	1248.29
380	…….	7.3421	…….	1411.78	…….	7.1865	…….	1319.13
390	....	7.5293	…….	1486.17	…….	7.3798	…….	1390.97
400	…….	7.7141	…….	1562.37	…….	7.5727	…….	1466.73
410	…….	7.8966	…….	1640.42	…….	7.7654	…….	1543.41
414.3	…….	7.9748	…….	1674.52	…….	7.8482	…….	1576.98
420	…….	8.0761	…….	1720.26	…….	7.9578	…….	1622.03
	
	Liquid phase							
200	a6.8	6.6360	1076	1064.60	4.83	4.8676	−110	−91.08
210	6.0	6.7178	1144	1131.37	5.16	5.1934	−58	−40.77
220	6.95	6.7996	1203	1198.96	5.50	5.5078	4	12.75
230	7.0	6.8814	1274	1267.36	5.80	5.8118	62	69.35
240	6.4	6.9632	1343	1336.58	6.10	6.1064	124	128.95
250	7.05	7.0450	1405	1406.62	6.37	6.3923	187	191.44
260	7.0	7.1268	1475	1477.48	6.65	6.6702	253	256.76
270	7.25	7.2086	1544	1549.16	6.92	6.9407	324	324.83
280	7.35	7.2904	1615	1621.66	7.19	7.2044	398	395.57
290	8.4	7.3722	1688	1694.97	7.45	7.4616	470	468.90
300	7.7	7.4540	1770	1769.10	7.74	7.7129	547	544.77
310	6.75	7.5358	1845	1844.05	8.00	7.9586	628	623.13
320	7.7	7.6176	1911	1919.82	8.22	8.1992	719	703.93
330	7.6	7.6994	2000	1996.40	8.45	8.4348	794	787.10
340	7.6	7.7812	2072	2073.80	8.69	8.6660	883	872.62
350	7.55	7.8630	2152	2152.02	8.90	8.8926	968	960.40
360	7.95	7.9448	2225	2231.06	9.12	9.1153	1062	1050.44
370	8.5	8.0266	2304	2310.92	9.34	9.3341	1154	1142.70
380	…….	8.1084	2392	2391.60	9.56	9.5492	1241	1237.12
390	…….	8.1902	…….	2473.09	…….	9.7610	…….	1333.68
400	…….	8.2720	…….	2555.40	…….	9.6693	…….	1432.32
410	…….	8.3538	…….	2638.53	…….	10.1746	…….	1533.06
414.3	…….	8.3890	…….	2674.52	…….	10.2619	…….	1576.98
420	…….	8.4356	…….	2722.48	…….	10.3768	…….	1635.80

a This column refers to temperatures 5° higher than indicated; e.g., 6.8 is the *C_p_* value at 205 °K.

**Table 3:** Analytical expressions for the thermodynamic functions of the ideal *CH*_2_-chain crystal and liquid above 200 °K, derived from *n*-paraffin data

Function	Solid phase (*T*≥200 °K)	Liquid phase (*T*≥200 °K)
		
*C_p_* (cal/mole °K)	0.0139 *T*+0.916+22.308×10^4^ *u*/*T*^2^	0.00818 *T*+5.00
*H–H*_0_ (cal/mole)	0.00695 *T*^2^+0.916 *T*−60+162.10*u*	0.00409 *T*^2^+5.00*T*−99
*S* (cal/mole °K)	0.0139 *T*+0.916ln *T*−3.94+(0.117789+162.10/*T)u*	0.00818 *T*+5.00ln *T*−23.26
*F–H*_0_ (cal/mole)	−0.00695 *T*^2^−0.916 *T*ln *T*+4.856 *T*−60−0.117789*Tu*	−0.00409 *T*^2^−5.00 *T*ln *T*+28.26*T*−99
	
	Liquid-solid difference (*T*≥200 °K)	
Δ*C_p_* (cal/mole °K)	−0.00572 *T*+4.84 −22.308×10^4^*u*/*T*^2^	
Δ*H* (cal/mole)	−0.00186 *T*^2^+4.84*T*−39−162.10*u*	
Δ*S* (cal/mole °K)	−0.00572 *T*+4.84ln *T*+19.32−(0.117789+162.10/*T*)*u*	
Δ*F* (cal/mole)	0.00186 *T*^2^−4.84 *T*ln *T*+23.404 *T*−39+0.117789 *Tu*	

*u*=exp[−3.32172 (414.3-*T*)/*T*]

## Footnotes

1 This means a crystal, large in all dimensions, of very long CH2 chain molecules, which contains no chain ends or branches and only an equilibrium number of defects.

2 Figures in brackets indicate the literature references at the end of this paper.

3 Actually, the specific heat data refers to *C*_satd_, the specific heat under the saturated vapor pressure of the material. For this work, the difference between *C_p_* and *C*_satd_ for the solid and liquid phases of the *n*-paraffins is negligible.

4 Even *n*-paraffins and odd *n*-paraffins are terms used here to mean *n*-paraffins containing an even or odd number of carbon atoms in the molecule.

5 See reference [3] for a further discussion of the above points.

6 We are not here concerned with the distinction between homophase premelting and impurity induced heterophase premelting.

7 In a private communication, J. P. McCullough has suggested that the nonlinearity in the liquid state is due to a contribution from short range order which decreases exponentially with temperature above the melting point. If this were the case, the liquid data would become more linear at higher temperatures. Higher temperature specific heat data can clarify this point.
